# Soil Microbial Functional and Fungal Diversity as Influenced by Municipal Sewage Sludge Accumulation

**DOI:** 10.3390/ijerph110908891

**Published:** 2014-08-28

**Authors:** Magdalena Frąc, Karolina Oszust, Jerzy Lipiec, Stefania Jezierska-Tys, Eucharia Oluchi Nwaichi

**Affiliations:** 1 Laboratory of Molecular and Environmental Microbiology, Department of Plant and Soil System, Institute of Agrophysics, Polish Academy of Sciences, Lublin 20-290, Poland; E-Mails: k.oszust@ipan.lublin.pl (K.O.); j.lipiec@ipan.lublin.pl (J.L.); stefania_tys@op.pl (S.J.-T.); 2 Department of Environmental Microbiology, University of Life Sciences in Lublin, Leszczyńskiego 7, Lublin 20-069, Poland; 3 Department of Biochemistry, Faculty of Science, University of Port–Harcourt, Port-Harcourt 5323, Nigeria; E-Mail: nodullm@yahoo.com

**Keywords:** sludge-born fungi, CLPP, molecular identification

## Abstract

Safe disposal of municipal sewage sludge is a challenging global environmental concern. The aim of this study was to assess the response of soil microbial functional diversity to the accumulation of municipal sewage sludge during landfill storage. Soil samples of a municipal sewage sludge (SS) and from a sewage sludge landfill that was 3 m from a SS landfill (SS3) were analyzed relative to an undisturbed reference soil. Biolog EcoPlates^TM^ were inoculated with a soil suspension, and the Average Well Color Development (AWCD), Richness (R) and Shannon-Weaver index (H) were calculated to interpret the results. The fungi isolated from the sewage sludge were identified using comparative rDNA sequencing of the LSU D2 region. The MicroSEQ® ID software was used to assess the raw sequence files, perform sequence matching to the MicroSEQ® ID-validated reference database and create Neighbor-Joining trees. Moreover, the genera of fungi isolated from the soil were identified using microscopic methods. Municipal sewage sludge can serve as a habitat for plant pathogens and as a source of pathogen strains for biotechnological applications.

## 1. Introduction

The increasing production of sewage sludge from wastewater treatment plants creates significant pressure on the management and disposal of this product [1,2]. Wastewater treatment plants generate large quantities of sludge that must be treated and reused or disposed of to protect the environmental and provide maximum benefits [3,4,5]. In Europe, an average sewage sludge dry weight production of 90 g per person per day results from primary, secondary and even tertiary treatment. Globally, it is believed that the sludge output will gradually increase in the coming decades due to urbanization and industrialization. According to data from the Polish Central Statistical Office, the amount of sewage sludge in 2010 amounted to 526,100 Mg d.m. Furthermore, according to the forecasts of the National Waste Management Program, the amount of sewage sludge in 2015 may reach 662,000 Mg d.m. in 2014 and may increase by an additional 64,000 Mg d.m. in the following three years [6]. Several disposal alternatives have been tried, including soil application, dumping at sea, landfilling and incineration [7]. Raw wastewater sludge contains more than 90% water with organic solids that cause problems in its transportation, treatment and disposal [8]. Therefore, sludge settling, dewatering and degradation are very important processes for wastewater sludge recycling and disposal [9]. Sewage sludge is composed of organic compounds and macro- and micronutrients and may contain organic micro-pollutants and pathogenic microorganisms [10,11].

Wastewater sludge contains a variety of microorganisms, such as viruses, bacteria, fungi, algae, protozoa and worms because it is a rich source of organic matter [8]. Microorganisms, mainly bacteria and fungi, play important roles in making nutrients available to plants [12]. Conventional disposal and sludge storage negatively influence the environment, especially regarding soil microbial activity, which is a very sensitive indicator of changes in the soil environment. Modifications in biological properties, including the microbial community, may precede detectable changes in the soil physical and chemical properties and provide early signals for improvement or degradation [13,14]. Community analysis using Biolog MicroPlates^TM^ was originally described by Garland and Mills [15], who inoculated Biolog MicroPlates^TM^ with a mixed culture of microorganisms to measure the community fingerprint and found that the characteristics of such a community could be assessed. In applied ecology research, community level physiological profiling (CLPP) was used as an assay for the stability of a normal population and to detect and assess changes in the variables that were introduced and the other anthropogenic and environmental factors [16,17,18]. CLPP, which is based on the ability of microorganisms to oxidize different carbon substrates, has been successfully used to differentiate between microbial communities from several habitats [19,20] and to detect modifications resulting from soil management and organic waste application [21,22,23].

Fungi are saprophytes that belong to a diverse group of organisms in the kingdom Eumycota [24] and have been used in wastewater sludge [8,25]. Several fungi that were previously isolated from these substrates are pathogenic to plants, animals and humans (e.g., mycoses [1]). In addition, studies regarding the fungal incidences in these environments are of hygienic, epidemiological and ecological significance. Sludge is increasingly being used to fertilize agricultural and forest areas and to reclaim devastated terrains. Thus, it is important to recognize the distribution of pathogenic fungal species in activated sludge [11]. However, only limited research is available in this area due to regulations in most countries regarding the permissible contents of heavy metals and the compulsory stabilization and disinfection of municipal sludge by treating with Lime, thermophilic digestion and pasteurization prior to land application [26]. Landfilling and the land application of sewage sludge are the most economical sludge disposal methods [2,27], and are necessary for monitoring the environmental state, including the soil microbial parameters. The aim of the study was to evaluate the influences of sewage sludge storage on the functional diversity of the soil microbial population and on the fungal diversity in sewage sludge.

## 2. Materials and Methods

### 2.1. Site Description and Sample Collection

The research site is located on Haplic Luvisols that were formed on heavy loamy sand with a 59% sand, 23% silt and 18% clay in the Eastern region of Poland (N51°17'16", E22°11'35"). The sewage sludge was sourced from a nearby municipal treatment plant at Nałęczów, Lubelskie, Poland. The municipal treatment plant is located in the protected zone of Kazimierski Landscape Park. This plant is surrounded by a health resort. Consequently, it is important to monitor the environmental state and study the influences of the waste on the soil microbial diversity to protect public health. All analyses were performed in 2011. The basic soil and sludge characteristics are given in Table 1. 

**Table 1:** Soil and sludge characteristics.

Parameter	Values	
	Soil	Sewage Sludge
pH	7.5	7.5
EC (ms m^−1^)	14.58	33.50
TOC %	0.845	30.00
N (g 100 g^−1^)	0.068	5.70
P (mg kg^−1^)	373.00	600.00
K (mg kg^−1^)	223.00	469.10
C/N	12.50	5.26
Ca (g kg^−1^)	6.38	2.52
Mg (g kg^−1^)	1.39	1.09
Na (g kg^−1^)	0.19	-
Zn (mg kg^−1^)	43.06	326.00
Cu (mg kg^−1^)	5.85	46.40
Cd (mg kg^−1^)	0.16	-
Cr (mg kg^−1^)	15.99	33.00
Pb (mg kg^−1^)	11.51	21.00

Notes: EC—electric conductivity, TOC—total organic carbon.

**Figure 1:**
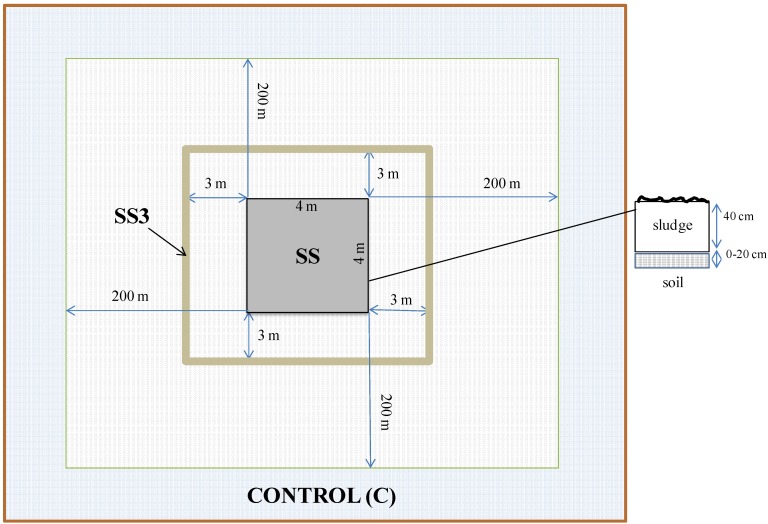
The research site near the Nałęczów sewage sludge landfill.

The study included three sites near the municipal treatment plant area. The following soils were subjected to analysis (Figure 1):

- a soil under the sewage sludge landfill (SS)—the landfill plot was a quadrat of 4 m by 4 m with 40 cm sludge layer on the soil surface,
- a soil located 3 m from a sewage sludge landfill (SS3), and
- a control soil (C) consisting of an undisturbed soil located 200 m from the sewage sludge landfill.

The soil samples were collected (0–20 cm depth) randomly, mixed, sieved through a 2-mm-mesh screen and stored at 4 °C until analysis. Municipal sewage sludge probes were collected from the plots where the sludge was stored. 

### 2.2. Chemical Analysis

The soil total organic carbon (Corg.) content was determined using the Tiurin method. The pH was measured from a soil aqueous extract using the electrometric method. The total N content was measured by using the Kjeldahl method and the total P content was measured by using the flow spectrophotometric method (SKALAR San System, Lilburn, GA, USA). The total K content was measured using the flame emission spectrometry method (AAS-3 Carl Zeiss Jena, Jena, Germany) after wet digesting the samples with sulfuric acid. The heavy metals were extracted using *aqua regia* and were measured using atomic absorption spectroscopy (AAS). The same methods were used for the soil and the sewage sludge.

### 2.3. Community Level Physiological Profiling (CLPP) Analysis

Community level physiological profiling was assessed using the Biolog EcoPlate^TM^ system (Biolog Inc., Hayward, CA, USA). Each 96-well plate consists of three replicates, with 31 sole carbon sources and a water blank. EcoPlates^TM^ were used to determine the carbon source use patterns of the SS, SS3 and C soil samples. The soils (1 g) were shaken in 99 cm^3^ of sterile water for 30 min before incubating for 20 min at 4 °C. After shaking, 120 µL of the sample suspension was inoculated into each well of the Biolog EcoPlates^TM^ and incubated at 26 °C. The utilization rate was indicated by the reduction of tetrazolium violet, a redox indicator dye that changes from colorless to purple [28]. The data were recorded at 590 nm every 24 h for up to 96 h. The microbial activity, which was expressed as the average well color development (AWCD), was determined for each microplate [20]. The data collected at 48 h were used to calculate the AWCD, the Shannon-Weaver (H) diversity indexes and the substrate richness (R), as shown by Gomez *et al.* [20]. In addition, the percentages of the carbon sources that were used by groups were evaluated.

### 2.4. Fungal Enumeration and Morphotypic Identification of Fungal Strains

The total number of fungi was determined by using the plate method with Bengal rose medium (Biocorp, Warsaw, Poland) and the serial dilution method. Soil (10 g wet weight) was treated with 90 cm^3^ of saline peptone water (Biocorp). Aliquots of the suspensions were transferred to petri dishes with the Bengal rose medium using pour plates. The plates were cultivated for 7 days at 26 °C. Fungal strains were isolated from the municipal sewage sludge and soil by using the same medium. The macroscopic variables that were analyzed included the colony diameter, conidia colors, obverse and reverse mycelium colors, degree of sporulation and the presence or absence of exudates [20]. Microscopic characteristics of the mycelia were examined using an optical microscope. Strains were identified at the genus level using microculturing growth on different media—Czapek agar and PDA (Biocorp). Cultures were incubated at 26 °C for 14 days. Genus identification was performed using the systematic classification methods of Domsch *et al.* [29], Burnett and Hunter [30] and Watanabe [31]. 

### 2.5. Molecular Identification of Fungi Isolated from Sewage Sludge

The most dominant fungi that were isolated from the sludge were identified using a molecular method. The method is based on rDNA-extraction and the subsequent amplification of the D2 large subunit region of the fungal rDNA [32] by using the Fast MicroSeq D2 LSU rDNA fungal sequencing kit (Applied Biosystems, Foster City, CA, USA). The fungal strains were grown on PDA medium (Biocorp). Preparation of the fungal DNA was performed with the PrepMan Ultra reagent (Applied Biosystems). A very small amount of one fungal colony was collected using tweezers and suspended in 200 µL of the PrepMan Ultra reagent. After incubation for 10 min at 100 °C, the suspension was mixed and centrifuged for 10 s allow the cell debris to settle. The supernatant solution was transferred to a new microcentrifuge tube and diluted at a ratio of 1:100 in deionized nuclease-free water [33]. A fast MicroSeq D2 LSU rDNA fungal PCR kit (Applied Biosystems) was used to amplify the D2 LSU rDNA region. The PCR was performed using the conditions listed in Table 2.

**Table 2:** PCR conditions.

Initial Step	Each of 30 Cycles		1 Cycle	Final Step
	Melt	Anneal	Final Extension	
HOLD	CYCLE		CYCLE	HOLD
95 °C	95 °C	64 °C	72 °C	4 °C
10 s	0 s	15 s	1 min	∞

The ExoSAP-IT® PCR Products Purification Kit for ABI (Affymetrix Inc., Santa Clara, CA, USA) was used to clean the PCR products before sequencing to eliminate any remaining labelled ddNTPs and for desalting. Next, 5 µL of the PCR product was mixed with 2 µL of the ExoSap reagent and incubated in a thermal cycling profile as follows: 37 °C for 15 min and 80 °C for an additional 15 min. The next step after PCR purification was to prepare the cycle sequencing. For this purpose, 13 µL of the forward/reverse mix from the MicroSeq D2 Fungal ID Sequencing Kit was used with 7 µL of the purified product. The cycling program is listed as follows: 96 °C for 1 min; (96 °C for 10 s; 50 °C for 5 s; 60 °C for 1 min 15 s) × 25. Next, the Performa Purification System (EdgeBio-Performa Gel Filtration Cartridges) was used and 20 µL of the reaction samples eluate was mixed with 20 µL of deionized formamide. Electrophoresis was run through an ABI 3130 ×l capillary sequencer (Applied Biosystems) with a 50 cm capillary array and polymer POP6_1. The MicroSEQ® ID software was used to assess the raw sequence files and to perform sequence matching to the MicroSEQ® ID (Applied Biosystems) to validated the reference database and to create Neighbor-Joining trees.

### 2.6. Statistical Analysis

The average well-color development, richness and the Shannon-Weaver index were statistically analyzed using an analysis of variance (ANOVA). The significance differences were determined using the Tukey test at *p* = 0.05. A principle component analysis was conducted on the optical density (OD) data from the 31 carbon sources following 48 h of incubation. Cluster analysis, including grouping of the treatments and features, was performed on the standardized data from the average absorbance values at 120 h. A dendrogram was prepared to present the similarity of the carbon utilization patterns of the substrates that were located on the Biolog EcoPlate^TM^ between the soil samples with scaled bond distances on the axis (Ward's method) and boundaries marked according to Sneath`s criteria (33% and 66%). The data were standardized according to AWCD in each microplate to remove the inoculum density effects [19]. All statistical analyses were performed using the Statistica 10.0 software (StatSoft Inc., Tulsa, OK, USA).

## 3. Results 

### 3.1. Microbial Activity

**Figure 2:**
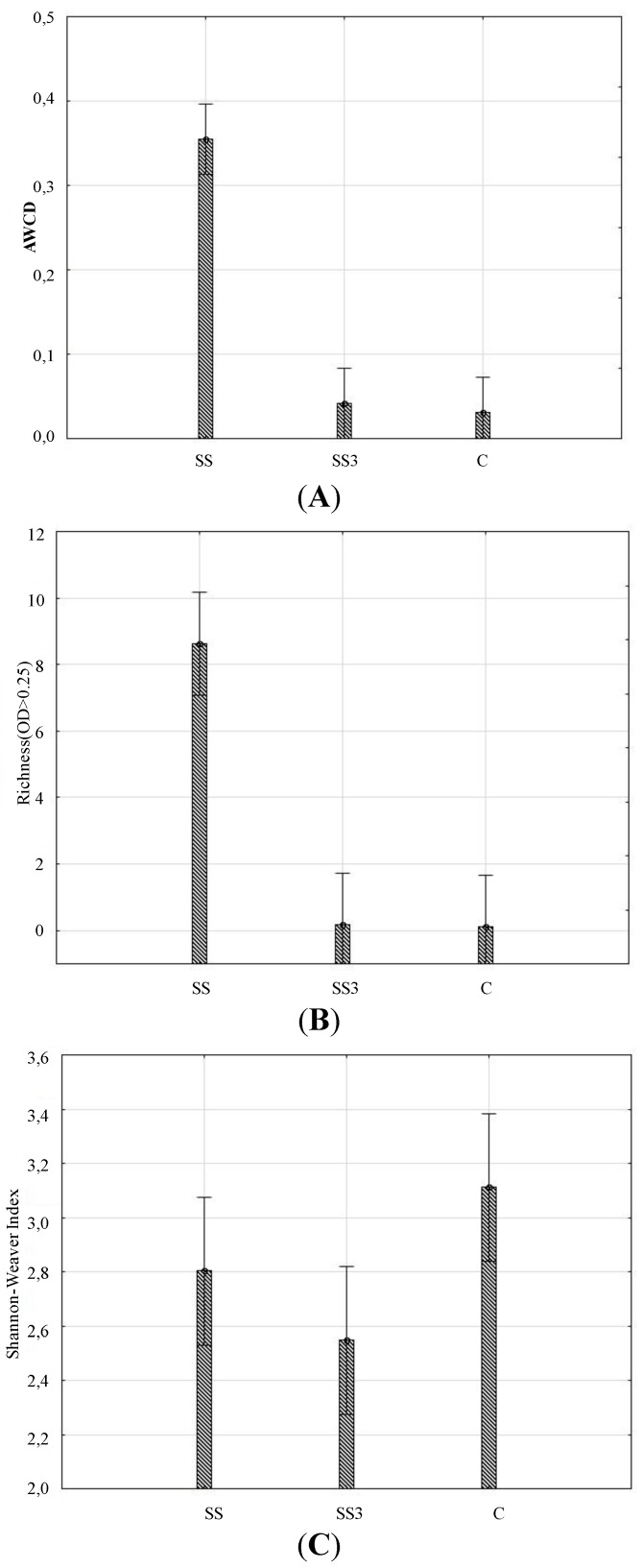
Average well-color development (AWCD) (**A**), Richness (**B**) and the Shannon-Weaver index (**C**) of the metabolized substrates in the Biolog EcoPlate in the soil. SS—soil under sewage sludge accumulation, SS3—soil 3 meters from the sewage sludge landfill, and C—control soil 200 meters from the sewage sludge landfill.

The results showed that the AWCD in the control soil was the lowest and increased under the SS3 and SS by 30 and 1000%, respectively (Figure 2A). In addition, the corresponding increases in R were 10 and 8000%. The AWCD and R were significantly greater for the SS when compared with the SS3 and C (Figure 2B). 

The lack of a significant difference (*i.e.*, the similar response) between the SS3 and C implies that the influential region of the organic inputs from the landfill regarding increases in microbial activity is characterized by the AWCD and the R is below 3 m. The impacts of sewage sludge on the catabolic potential of microorganisms only occur where the sewage sludge is stored. The average utilization (*i.e.*, AWCD) of the C sources increased with incubation time for all treatments (data not shown). The Shannon-Weaver index was the highest in the control soil, but was not significantly different from the other treatments (Figure 2C). This result indicates that the microbial populations were stable in the soils.

**Figure 3:**
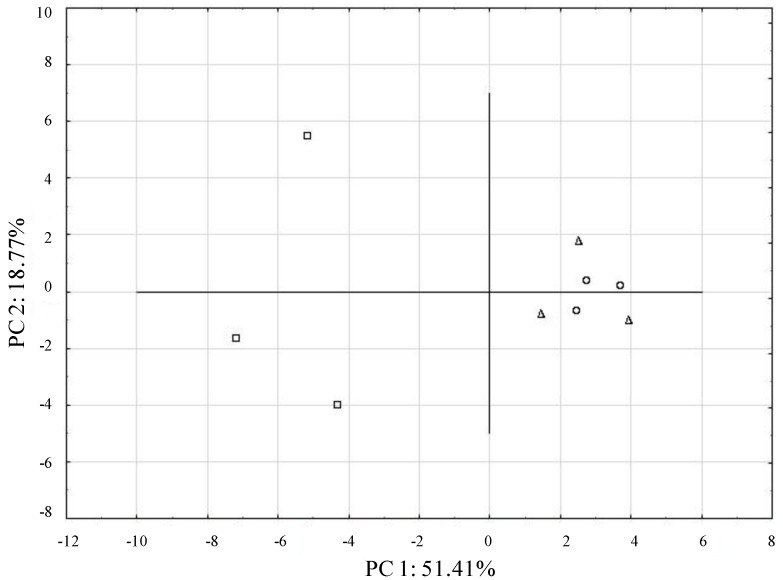
Principal component analysis of the carbon source activity data in Biolog EcoPlates, from the control soil □, the soil under sewage sludge accumulation Δ, and the soil 3 meters from the sewage sludge landfill ○.

To determine the extent of treatments differentiation with regard to the metabolism of the carbon sources, a principal components analysis was performed. The first and the second principal components (PC1 and PC2) explained 51.41% and 18.71% of the data variance, respectively. The control soil and the soil that was 3 m from the sewage sludge landfill were clustered together (C and SS3) and were different from the soils under sewage sludge accumulation (Figure 3).

The carbon sources were significantly correlated with PC1 and PC2 (R > 0.7) (Table 3). Several substrates, such as pyruvic acid methyl ester, tween 80, α-d-lactose, d-mannitol, N-acetyl-d-glucosamine, dl-α-glycerol phosphate, d-galactonic acid γ-lactone, 4-hydroxybenzoic acid, d-malic acid and l-arginine were intensively metabolized by the microbial communities from the soil under the sewage sludge accumulation. The microbial communities from the control and the soils that were collected 3 m from the sewage sludge landfill intensively used tween 40 and N-acetyl-d-glucosamine. The microbial communities from the SS3 soil used i-erythritol and control soil used d-cellobiose. Optical densities of less than 0.25 (threshold for positive response) were recorded for glycogen and β-methyl-d-glucoside for all samples. 

**Table 3:** Carbon substrates utilized by the microorganisms in Biolog EcoPlate^TM^ that were significantly correlated to PC1 and PC2 (R > 0.70).

	Principal Components	
PC1		PC2
d-Galactonic Acid	N-Acetyl-d-Glucosamine	d-Xylose
l-Arginine	d-Glucosaminic Acid	Cyclodextrin
Pyruvic Acid Methyl Ester	Itaconic Acid	α-A-d Lactose
i-Erythritol	Glycyl-l-Glutamic Acid	Putrescine
2-Hydroxybenzoic Acid	d-Glucose-1-Phosphate	
Tween 80	α-Ketobutyric Acid	
d-Mannitol	Phenylethylamine	
4-Hydroxybenzoic Acid	dl-α-Glycerol Phosphate	
l-Serine	d-Malic Acid	

**Figure 4:**
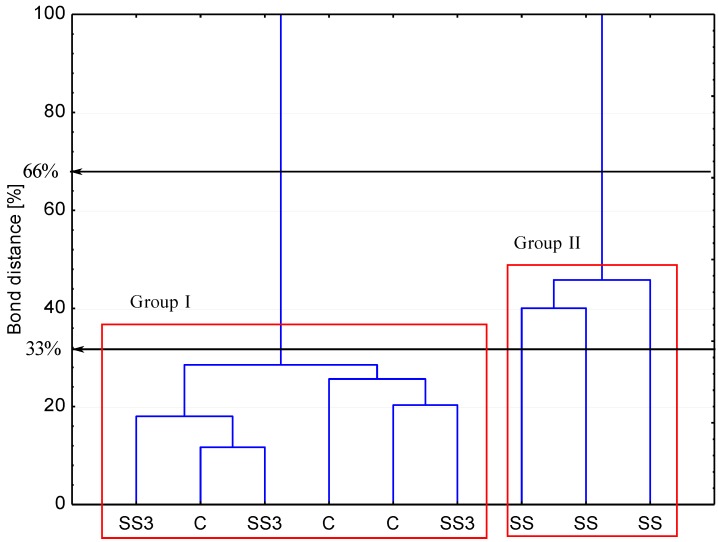
Dendrogram of the bond distances between the carbon utilizations patterns of the substrates located on the Biolog EcoPlates^TM^. Grouping was conducted according to the stringent Sneath criterion (33%) and the less restrictive criterion (66%), respectively. SS—soil under sewage sludge accumulation, SS3—soil 3 meters from the sewage sludge landfill, C—control soil 200 meters from sewage sludge landfill, *n* = 3.

Figure 4 presents the bond distances between the tested objects, where clustering was evaluated according to the carbon utilizations patterns of the substrates located on the Biolog EcoPlates^TM^. 

**Figure 5:**
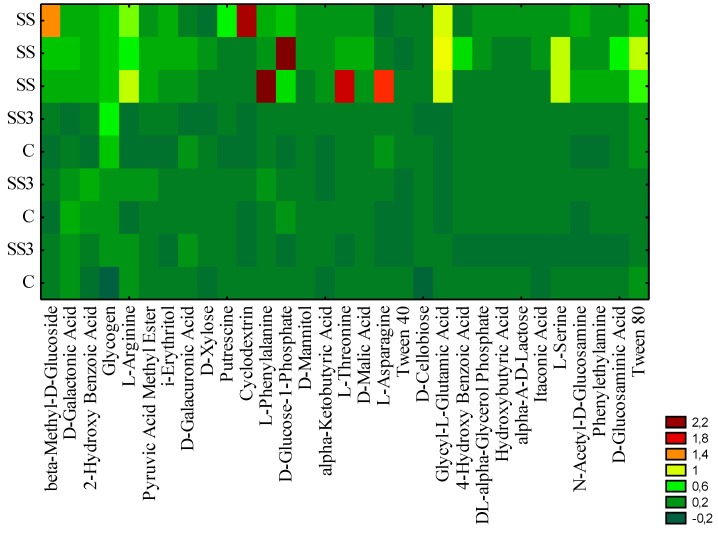
Biolog EcoPlate^TM^ carbon source utilization intensity diagram. SS—soil under sewage sludge accumulation, SS3—soil 3 meters from the sewage sludge landfill, C—control soil 200 meters from sewage sludge landfill, *n* = 3.

A similar response occurred between the microbial communities under the SS3 and the control (group 1) towards carbon substrate utilization. This findings were confirmed by the carbon substrate utilization intensity diagram (Figure 5). Group 1 (C and SS3) and group 2 (SS) differed regarding their use of particular carbon sources. A better aptitude to substrate utilization was revealed by the SS than by the C and SS3. The most intensively used substrates by the SS community included the following: β-methyl-d-glucoside, cyclodextrin, l-phenylalanine, d-glucose-1-phosphate, l-threonine, and l-asparagine.

**Figure 6:**
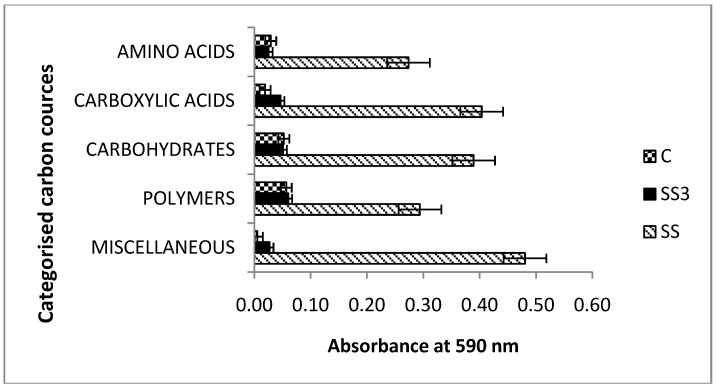
Categorized substrate utilization patterns by the microbial communities from the soil after 48 h of incubation. Errors bars indicate the standard errors of the mean (*n* = 3).

Significant differences were observed in the categorized substrate utilization patterns among the microbial communities of the three soil samples (Figure 6). The carboxylic acids, carbohydrates and miscellaneous groups were the most intensively metabolized, especially in the soil under sewage sludge accumulation.

### 3.2. Pathogenic Fungi and Yeast

**Figure 7:**
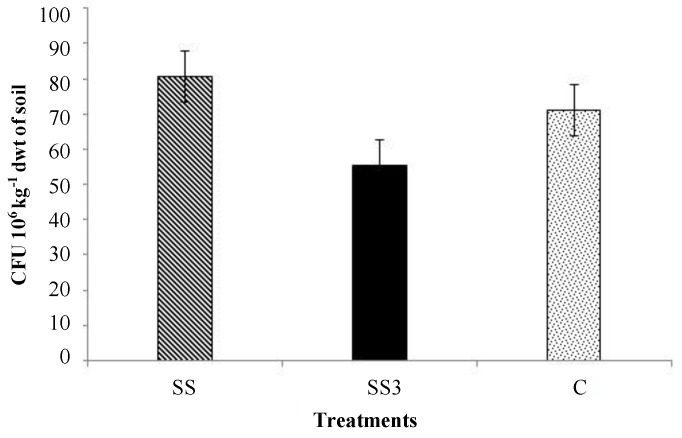
Mean values of colony forming units (CFU) of the fungal communities.

The effects of sewage sludge accumulation on the total number of fungi are presented in Figure 7. Mean values of colony forming units were greater in the soil under sewage sludge accumulation (80.43 × 10^6^ CFU kg^−1^) relative to in the control (71.20 × 10^6^ CFU kg^−1^) and the SS3 soil (55.54 × 10^6^ CFU kg^−1^). 

**Figure 8:**
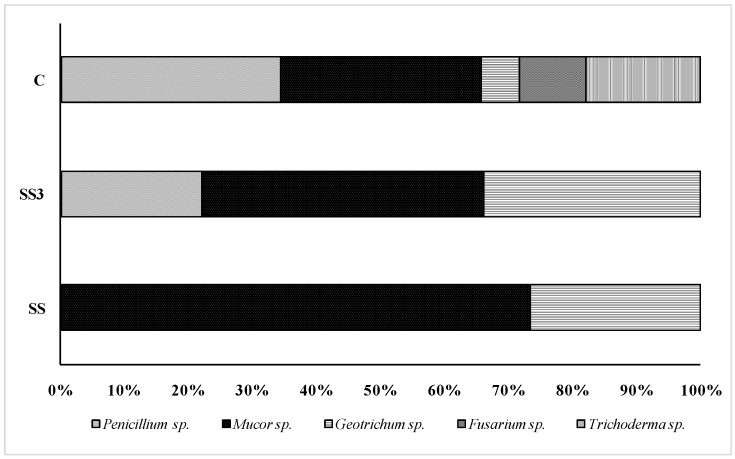
Relative distribution of fungal communities in particular soils: control soil (C), soil under sewage sludge accumulation (SS) and soil 3 m from the sewage sludge landfill (SS3).

The most common genera of fungi that were recorded in each sample are shown in Figure 8. Plant pathogenic fungi (*Mucor*) and yeast (*Geotrichum*) prevailed in the soil under sewage sludge accumulation and in the soil that was 3 m from the sewage sludge landfill (Figure 8). 

In the control soil, different species from the *Penicillium* and *Mucor* genera were isolated frequently. We observed a higher fungal diversity in the control soil than in the soil under sewage sludge accumulation.

**Figure 9:**
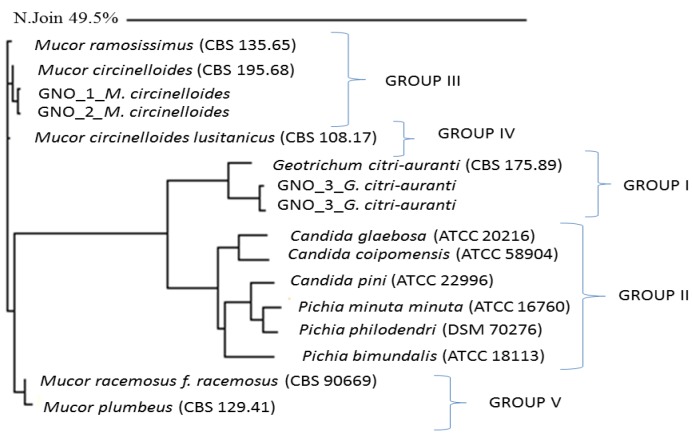
Neighbor-Joining phylogenetic tree showing the relationships between fungi isolated from municipal sewage sludge and related fungi in the MicroSeq database based on the sequences of the D2 LSU region of nuclear rDNA.

Molecular identification of the dominant fungi that were isolated from the sewage sludge and the phylogenetic analysis of the DNA sequences showed that *Mucor circinelloides* and *Geotrichum citri-aurantii* were present in settled sludge as a fungal monoculture (Table 4, Figure 9). Phylogenetic analyses of the LSU D2 rDNA nucleotide sequences demonstrated that the fungal monoculture was created by molds from the *Mucor* and *Geotrichum* genus. The first group includes molds, and the second group includes yeast species. An analysis of the phylogenetic tree indicated a 98.13% phylogenetic relationship with *M. circinelloides*. In addition, a very close relationship with *M. ramosissimus* (96.26%) was observed. Furthermore, *M. racemosus* and *M. plumbeus* were closely related to the fungal isolates from the sewage sludge. The genetic distances between the above-mentioned species and the environmental fungi were 6.54% and 8.41%, respectively. The analysis of the second group indicated that the closest phylogenetic relationships were observed between the isolates from the sewage sludge and *G. citri-aurantii* (93.46%). A higher genetic distance was observed for the different yeast genera of *Candida* and *Pichia* with 16.81% (83.19% similarity) and 23.35% (76.65% similarity), respectively (Figure 9). 

**Table 4:** Molecular identification of fungi isolated from municipal sewage sludge.

Sample Name	Specimen Score	Top Match	% Match	Consensus Length	Library Entry Length
GNO_1_M_circinelloides	44	*Mucor circinelloides* (CBS = 195.68)	99.62	370	384
GNO_2_M_circinelloides	40	*Mucor circinelloides* (CBS = 195.68)	99.30	384	384
GNO_3_G_citri-aurantii	42	*Geotrichum citri-aurantii* (CBS = 175.89)	93.12	271	271
GNO_4_G_citri-aurantii	42	*Geotrichum citri-aurantii* (CBS = 175.89)	92.42	271	271

## 4. Discussion

### 4.1. Microbial Activity 

The preliminary results indicated that municipal sewage sludge containing organic matter and macronutrients (N, P and K) and the permissible values of heavy metals could be useful for improving microbial activity and soil fertility. A stimulating effect of the sludge on the microbial activity and the oxidative capacity developing in the Biolog EcoPlates^TM^ was clearly shown based on the higher AWCD and Richness in the SS soils relative to the SS3 and C soils. Frąc *et al.* [23] suggested that in the increased diversity indices after applying organic matter potentially resulted from the development of different microbiota. In agreement with our results, previous studies have demonstrated microbial communities with a high functional potential for carbon utilization in dairy sewage sludge [23], vermicompost, manures [20], and straw [34].

As shown in previous studies, the application of sewage sludge improves the soil structure [35] increases the resistance of the soil to erosion [36], or plays a significant role in the natural remediation of metals and aromatic compounds [37]. Besides stimulating microbial activity, these effects can be helpful for controlling erosion from the erodible loess soils that were used in this study [38]. 

Changes in the categorized substrate utilization patterns among the microbial communities in the studied treatments suggest that the incorporation of sewage sludge may stimulate microbial carbon use and metabolism in the soil. Similar results after the application of organic matter to the soil were observed [28]. The principal components analysis was used to define the different carbon substrate uses that were metabolized the most intensively. According to Gomez *et al.* [22], the carbon sources in the Biolog^TM^ assay provide a wide set of compounds that can be used to estimate the relative potential metabolic versatility.

### 4.2. Pathogenic Fungi and Yeast

The number of microscopic fungi in sewage sludge and soil under sewage sludge accumulation is usually relatively high. As reported previously, mean values of colony forming units in the same experimental site ranged from 55.54 × 10^6^ to 80.43 × 10^6^ CFU kg^−1^ of soil dry matter (Figure 5). Microscopic fungi play an important role in the decomposition of sludge organic matter [39]. In the present study, the fungal population increased (but not significantly) in the soil under sewage sludge accumulation. A similar effect was observed by Brendecke *et al.* [40]. The short-term increase in the fungal population that occurred as a function of the addition of sewage sludge may be explained by the presence of easily degradable organic components in the waste.

A molecular analysis of the fungi that were isolated from the municipal sewage sludge indicated that this sludge could be dangerous for plants due to the presence of *M. circinelloides* and *G. citri-aurantii*. These fungal species are common plant pathogens [41,42]. Worldwide citrus sour rot, which is caused by *Geotrichum citri-aurantii*, has been reported as an important postharvest disease in citrus fruit [42]. In addition, *M. circinelloides* causes disease in fruit [41] and tomatoes [43]. However, fungi such as *M. circinelloides* are also of biotechnological interest as a source of carotenes, proteins and lipids because they accumulate high levels of these compounds in their mycelium [44,45]. Moreover, *M. circinelloides* lipids have gained attention because they can be easily converted into biodiesel, suggesting that the biomass of this fungi could be used as an alternative to plant oils as a feedstock for biodiesel production [46,47].

Microscopic identification of the fungi indicated that the control soil had the highest fungal diversity. However, the soil that was collected from the plots with sewage sludge accumulation were nearly “fungal monocultures” (Figure 6), as in the sewage sludge. This result suggests that the fungal species from the sludge won the competition for the soil fungi niche. All of the fungi that were isolated from the soil under sewage sludge accumulation or at 3 m from the sewage sludge landfill were closely related to *Mucor* or *Geotrichum*. Detecting the plant pathogenic fungi (*Mucor*) and yeast (*Geotrichum*) application limits of the sludge without thermal treatment and such use should be avoided, and the fungal and yeast compositions should be monitored before applying sludge in the soils and during the storage of this waste. Pascual *et al.* [1] reported that the impacts of sanitized (Autothermal Thermophilic Aerobic Digested, ATAD) and non-sanitized (Anaerobic Mesophile Digested, AMD) sewage sludge on the activities and functional diversities of the soil microbial communities and plant growth were similar. However, sanitation can be beneficial under conditions in which the pathogens migrate to the aquatic environment (the plants do not migrate). The changes in the fungal composition potentially resulted from the improved conditions that favored fungal growth. Because the application of non-stabilized sludge on the land or its storage may result in serious hygienic problems, our knowledge regarding sludge borne-fungal compositions is significant from a practical point of view. Fungi are opportunistic organisms that possess potential pathogenic properties for humans, animals and plants. However, some fungi have higher pathogenic potentials than others. To minimize human infection, it is necessary to monitor the mycological quality of municipal sewage sludge. From a biotechnological point of view, sewage sludge can be one source of enzymes or microorganisms that produce enzymes and different metabolites. Enzymes that are secreted or associated with these microorganisms are vital for the rate-limiting step of anaerobic digestion, hydrolysis [48]. The activities of these hydrolases are important for establishing the overall limiting rate of the processes. This rate limiting step involves the breakdown of large polymeric substrates, such as carbohydrates (cellulose, starch, proteins), into small and low molecular weight compounds (glucose, amino acids, *etc.*) that are capable of entering the microbial cells and participating in its cellular metabolism. 

## 5. Conclusions

1. This study showed that community-level physiological profiling using Biolog EcoPlates^TM^ can be used as a sensitive and effective indicator for evaluating microbial functional diversity and microbial communities under sewage sludge accumulation. These parameters can be useful for monitoring the soil microbial state following sewage sludge application.
2. Municipal sewage sludge is rich in molds and yeasts, which are known as fungal plant pathogens. Most fungi that were recovered in this investigation can be considered as potential pathogens. Therefore, all sewage sludge research and accumulation workers should be careful to avoid mycotic infections, and their production must be adapted to control the spread of pathogenic fungi in the environment.
3. Fungi in sewage sludges are not only pathogens but also have practical uses for the development of biopreparations for environmental technologies and agricultural biotechnology, especially for the disposal and recycling of organic wastes. Future research studies are needed to assess the properties and use of fungal strains that are isolated from sludges and soils during organic waste degradation.
